# High abundance of ArfGAP1 found in the mossy fibers in hilus of the dentate gyrus region of the mouse brain

**DOI:** 10.1371/journal.pone.0189659

**Published:** 2017-12-14

**Authors:** Sergiy Chornyy, Anna Parnis, Michael Shmoish, Dan Cassel

**Affiliations:** 1 Department of Biology, Technion-Israel Institute of Technology, Haifa, Israel; 2 Bioinformatics Knowledge Unit, Lorry I. Lokey Interdisciplinary Center for Life Sciences and Engineering, Technion-Israel Institute of Technology, Haifa, Israel; University of Modena and Reggio Emilia, ITALY

## Abstract

The Arf GTPase-activating protein ArfGAP1 and its brain-specific isoform ArfGAP1^B^ play an important role in neurotransmission. Here we analyzed the distribution of ArfGAP1 in the mouse brain. We found high levels of ArfGAP1 in the mouse dentate gyrus where it displayed especially elevated level in the polymorph layer (hilus). Importantly, the ArfGAP1 signal follows the pathway of the granular cell axons so-called mossy fibers which extend from the dentate gyrus to CA3 via stratum lucidum and partially stratum oriens. Additionally, we identified differential expression of ArfGAP1 in the isocortex. Thus, staining with anti-ArfGAP1 antibodies allows distinction between cortical cell layers 1, 2, 3 and 5 from 4 and 6. Taken together, our data suggest that ArfGAP1 can be used as a specific marker of the dentate mossy fibers and as for visualization of cortical layers in immunohistochemical studies.

## Introduction

ADP-ribosylation factor GTPase-activating protein 1 (ArfGAP1) is a well-known regulator of vesicle cycling through the Coat Protein Complex I (COPI) system that mediates retrograde Golgi to ER transport [1]. The ArfGAP1 protein is ubiquitously expressed in many tissues with the highest level found in liver and brain. Our previous studies demonstrated that the major ArfGAP1 isoform in the rat brain (ArfGAP1^B^) contain an in-frame insertion of 10 amino acids after residue 239, and a deletion of 22 amino acids after ArfGAP1 residue 259. These insertion/deletion regions might function as alternative Golgi localization determinants [2] or may have yet undetermined regulatory functions in the brain.

Protein trafficking in neurons plays essential roles in the establishment and maintenance of cell morphology and synaptic function. Recent findings indicated that ArfGAP1 plays a role in trafficking in neurons that may differ from its function as a COPI regulator. Thus, it was found that ArfGAP1 can regulate trafficking and targeting of the GABA transporter-1 in the axonal terminals [3]. More recently, ArfGAP1^B^ was found to function as a regulator of neurotransmission via the formation of tripartite AP-1/σ1A-ArfGAP1-Rabex-5 complexes [4]. Taken together, the aforementioned research suggests that ArfGAP1^B^ might be an essential regulator of a variety of brain functions including nerve cell secretion, signaling, neurotransmission and neurodegeneration. Here, we carried out a morphological study aimed at revealing how ArfGAP1 is distributed among different regions of the mouse brain.

## Materials and methods

### Animals and sample preparation

Experiments involved mouse brain tissues preparations were conducted using brains extracted from corps of eight-week-old naïve adult males CD1 mice, which had not received any special treatment or stress, and kindly donated for our investigation by Prof. Cohen, Technion, Biology Department, Haifa, Israel. Prior performing our experiments, we had consulted with the Institutional Animal Care and Use Committee (IACUC) of the Technion, Haifa, Israel and confirmed that a special approval for the usage of mice corpses was not required. All ethic procedures involving housing, handling and euthanizing animals were carried out in the Laboratory of Prof. Cohen in accordance with their IACUC approved protocol. After brains were removed from the skull, they were washed with ice-cold phosphate-buffered saline (PBS) followed by 4% paraformaldehyde fixation and overnight incubation in 30% sucrose in PBS. Brains were stored in OCT (Tissue-Tek, Netherlands) at -20°C and sectioned on a CM1860cryostat (Leica biosystems, Germany) at 30 μm followed by immunohistochemical processing. For protein analysis, several brain regions were immediately isolated from mice brains without fixation and carefully cleaned in ice-cold PBS followed by protein extraction.

### Immunohistochemistry

Brain tissue sections were washed in ice-cold PBS, incubated in 1% NaBH4 for 15 min to decrease autofluorescence, and permeabilized in TBST (PBS with 0.3% v/v Triton X-100) for 30 min followed by incubation for 2 hrs in blocking buffer containing 5% w/v bovine serum albumin (BSA, Sigma-Aldrich), 5% v/v goat serum (Sigma-Aldrich) and 5% w/v normal donkey serum (Jackson ImmunoResearch) in PBST. Sections were immunostained with rabbit anti-ArfGAP1 (1:400, ab183746, AbCam, UK), rabbit ArfGAP1 antibodies against the insertion peptide that is present in the brain/heart isoforms previously generated by us [2] (1:400), mouse anti-ZnT3 (1:100, Synaptic Systems, France), mouse anti-GFAP (1:500, Millipore, USA) overnight at 4°C, washed with PBS and incubated for 1 hr with appropriate secondary antibodies (1:400, Alexa-488 conjugated anti-rabbit and CY3 conjugated anti-mouse from Jackson Immuno Research, USA). Sections were then washed in PBS, incubated with DAPI (1 mg/ml, Sigma-Aldrich) for 5 min or NeuroTrace™ 530/615 for 2 hrs (1:50, Thermofisher Sci) followed by washing in water, and mounted on glass slides. Images were acquired using a Nicon A1-R confocal microscope for whole-brain mapping of ArfGAP1 localization (coronal view, bregma -1,94 mm and sagittal view, lateral 1.68 mm) and a Zeiss LSM 700 laser scanning confocal microscope (Germany) for more detailed analysis (coronal view bregma -1.94) at 10X.

### Western blot assay

Proteins were extracted from 100 μg of brain tissue after homogenization using 100 μl of 20mM Tris-HCl buffer (pH 7,5), sonicated (Misonix Sonicator, USA), centrifuged and protein concentration in the supernatant was assayed using the Nano Drop ND-1000 spectrophotometer (Thermo Fisher Scientific, USA). Aliquots of the extract containing 50 μg protein were separated by reducing SDS-PAGE (10%) and electroblotted onto nitrocellulose membranes. The blots were blocked by using 0,2% EZ-block solution (Biological Industries, USA) in PBS Tris-buffered salt with Tween-20 (PBST). The blots were incubated overnight at 4°C with rabbit anti-ArfGAP1 antibody (1:1000, ab183746, AbCam, UK) and rabbit ArfGAP1 antibodies against the insertion peptide that is present in the brain/heart isoforms previously generated by us [2](1:3000), then incubated in goat-anti-rabbit secondary antibody-conjugated horseradish peroxide (1:2500, Sigma-Aldrich). Proteins were visualized by chemiluminescence using the Clarity Western ECL Substrate (Bio-Rad Laboratories, USA). To ensure even loading of the samples, the same membrane was probed with rabbit anti-GAPDH antibody (1:1000, AbCam). The analysis was performed by Image Quant LAS 4000 (GE HealthCare Life Science, USA). The intensity of the ArfGAP1 band in each sample was normalized relative to GAPDH.

### Statistical analysis

Linear regression analysis was performed using Excel (Microsoft). Quantification of Western Blot data was conducted using Prism (GraphPad, USA). Data correspond to means ± SEM, n = 3.

## Results and discussion

**Fig 1:**
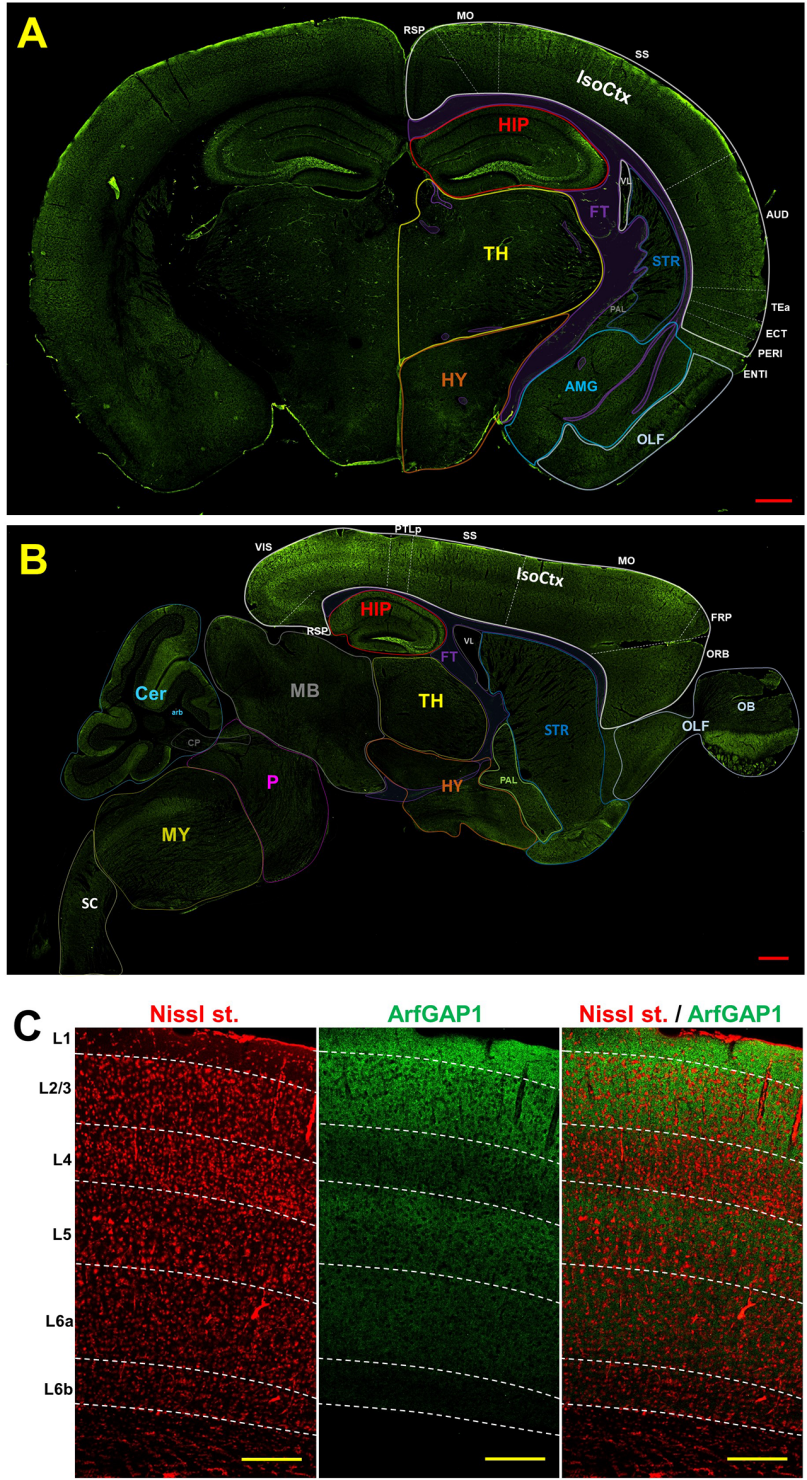
Mapping of ArfGAP1 localization in the adult mouse brain. A. Coronal plane. B. Sagittal plane. C. Double labeling of ArfGAP1(green) and Nissl st. (stain) (red) of cortical layers (L). IsoCtx–isocortex, RSP–retrosplenial cortex, VIS–visual cortex, PTLp–posterior parietal association areas, MO–motor cortex, SS–somatosensory cortex, AUD–auditory cortex, TEa–temporal association areas, ECT–ectorhinal cortex, PERI–perirhinal cortex, ENTI–entorhinal cortex, FRP–frontal pole, ORB–orbital area, OLF–olfactory cortex, OB–olfactory bulb, STR–striatum, AMG–amygdala, FT–fiber tracts, TH–thalamus, HY–hypothalamus, HIP–hippocampus, VL–lateral ventricle, PAL–pallidum, MB–midbrain, P–ponce, MY–medulla, Cer–cerebellum, arb–arbor vitae, CP–cerebral peduncle, SC–spinal cord. The scale bars represent 500 μm (A,B) and 200 μm (C).

**Fig 2:**
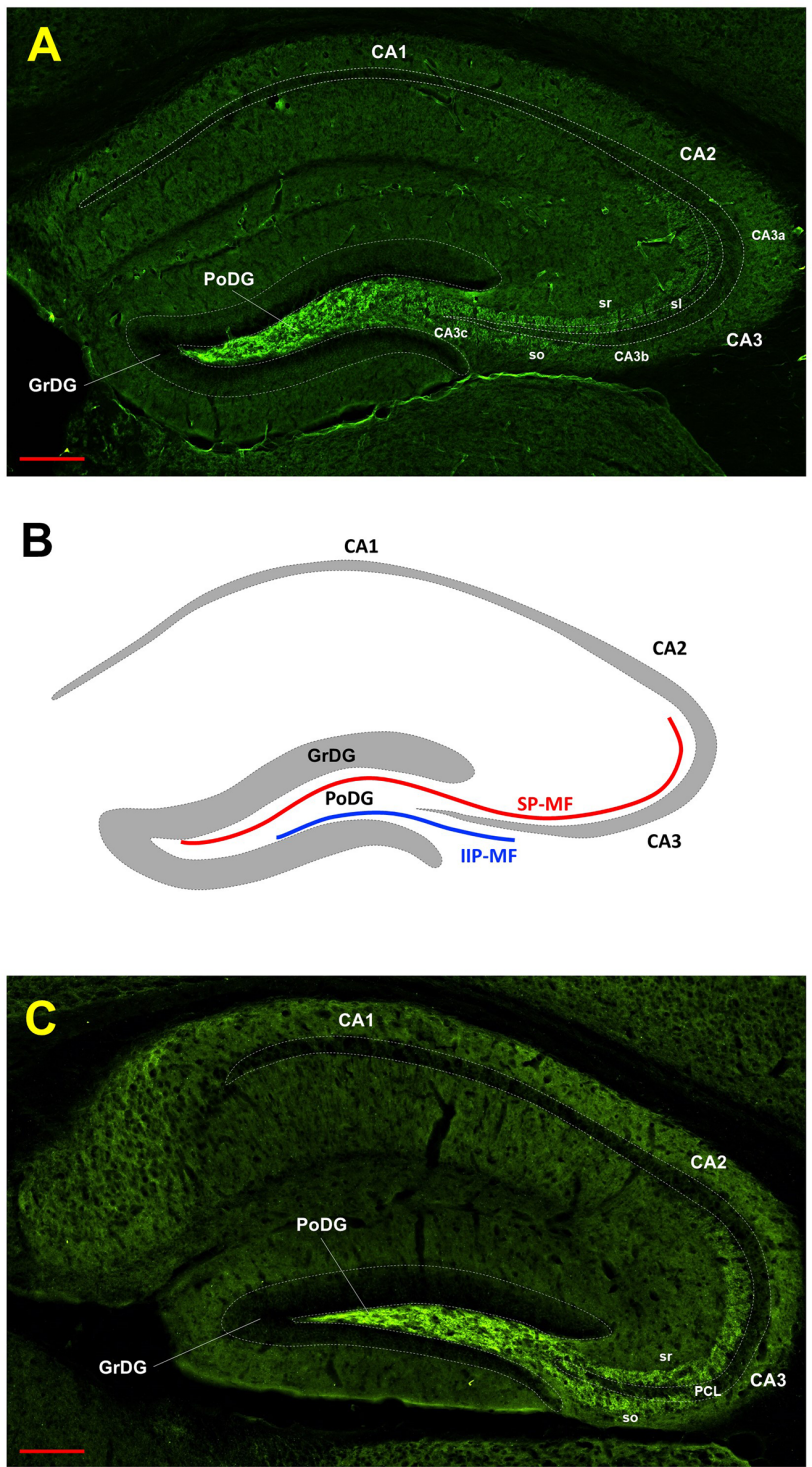
ArfGAP1 immunoreactivity in the hippocampus. A. Coronal plane. B. schematic view of the mossy fibers projections. C. Sagittal plane. SP-MF–suprapyramidal bundle and IIP-MF–intra- and infrapyramidal bundles of the mossy fibers projections. C. Sagittal plane. poDG–polymorph layer (hilus) of the DG, grDG–granular cell layer of the DG, sr–stratum radiatum, slu–stratum lucidum, so–sorstratum oriens. CA1, CA2 and CA3 –Cornu Ammonis of the hippocampus. The scale bars represent 200 μm.

To identify the ArfGAP1 distribution among different mouse brain regions, we performed an immunohistochemical analysis of the mouse brain using a commercial antibody recognizing all ArfGAP1 variants (ab183746, AbCam). Examination of the mouse brain showed high abundance of ArfGAP1 in the dentate gyrus (DG) and moderate level in the isocortex (IsoCtx) compared to other regions (Fig 1A and 1B). The ArfGAP1 levels looked similar in both hemispheres. However, ArfGAP1 was not detected in most of the areas were the fibers tracts are spanning. Within the DG, ArfGAP1 displayed especially elevated level in the polymorph layer (hilus) and was absent in the granule cell bodies (Fig 2). Interestingly, the ArfGAP1 signal follows the pathway of the DG granule cell axons so-called mossy fibers. The mossy fibers extend from the DG to CA3c and partially CA3b areas through suprapyramidal bundle in stratum lucidum together with intra- and infrapyramidal projection located mainly in stratum oriens (Fig 2B). We identified these projections in both coronal and sagittal brain tissue sections (Figs 1 and 2).

In the IsoCtx, we found diverse levels of ArfGAP1 immunoreactivity. The most intense staining among all areas was observed in the sagittal plane such as the visual and posterior parietal association areas (PTLp) that have higher abundance compare to the somatosensory (SS) and somatomotor (MO) areas (Fig 1B). In contrast, the other areas of the IsoCtx have low level of abundance. In addition, we detected separation of ArfGAP1 staining in the cortical layers. Thus, layers 1, 2, 3 and 5, have higher signal than layers 4 and 6a, while the layer 6b has an absent or very low immunoreactivity of ArfGAP1 as seen in both the coronal and sagittal planes (Fig 1A and 1B) and using Nissl staining (Fig 1C).

Functionally, layers of the IsoCtx can be divided into three parts: the supragranular layers 1–3, the internal granular layer 4 and the infragranular layers 5 and 6 [5]. Layer 1 has very few neurons and cells and are mainly composed of dendrites and axons that extend from lower levels of the IsoCtx, while layer 2 contains granular cells and small pyramidal cells. Glanule cells and transverse fibers are most prominent in layer 4. In contrast, layers 3 and 5 consist mostly of pyramidal cells. Moreover, layer 5 contains giant pyramidal cells (Betz cells) which are the largest excitatory projection neurons in the brain that can project to the spinal cord and form synapses to target muscles that control movement. Layer 6 contains less common cell types, including horizontal cells, fusiform cells and the cells of Martinotti [5]. There are many staining techniques to analyze neuronal morphology and cytoarchitecture of the IsoCtx and the whole brain including conventional techniques such as Nissl, Golgi and Weigert stainings [6, 7]. However, there are difficulties in the identification of some layers using conventional techniques. Recent experimental approaches including in situ gene expression analysis allow well-defined morphological identification of the specific cortex layers (reviewed by Molyneaux et al, 2007 [8]). Using anti-ArfGAP1 antibodies we were also able to obtain a clear visualization of the structural layers of the IsoCtx (Figs 1 and 2). Taken together, our data suggest that the ArfGAP1 antibody can be used for visualization of the hilus of the DG including its projections to CA3 region and cortical layers in immunohistochemical studies.

**Fig 3:**
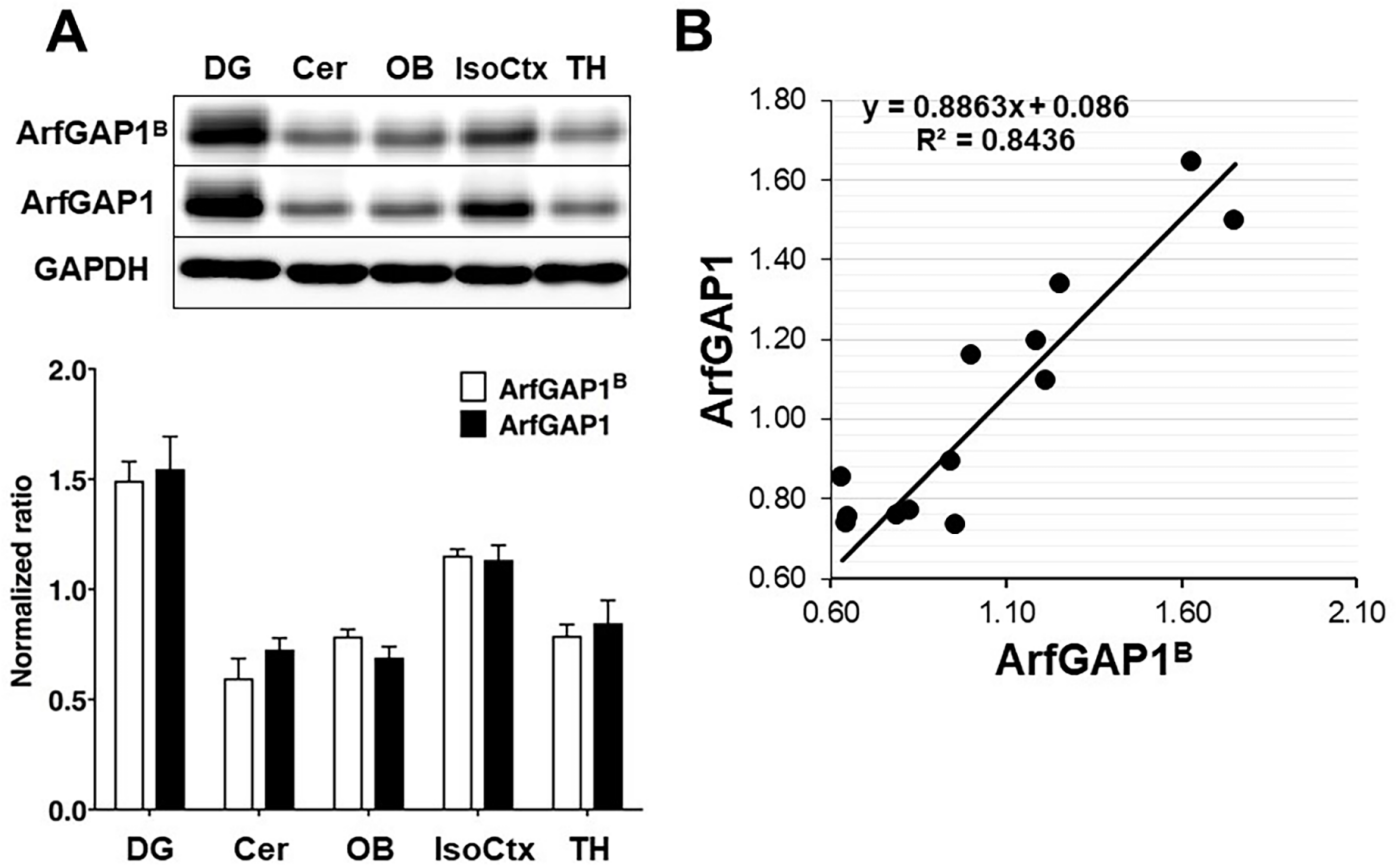
Western blot detection of total ArfGAP1 and ArfGAP1^B^ in adult mouse brain regions. A. Data were normalized to GAPDH protein levels for each region. DG–dentate gyrus, Cer–cerebellum, OB–olfactory bulb, IsoCtx–isocortex, TH–thalamus. Data expressed as the mean ± SEM, n = 3. B. Linear regression analysis of the pairs of data obtained using the two antibodies from each tissue in each of the 3 experiments.

We attempted to determine the distribution of the brain-specific ArfGAP1 isoform using a previously-developed antibodies directed against the ArfGAP1^B^ insertion peptide [2]. However, these antibodies were found inefficient in immunohistochemical staining and produced a high background. As an alternative, we performed Western Blot analysis on isolated brain regions using the antibodies recognizing all forms of ArfGAP1 or the B isoform only [2, 4] (Fig 3A). Both antibodies equally detected the highest level of proteins in the DG, moderate level of proteins in the IsoCtx and the lowest levels in the cerebellum (Cer), thalamus (TH) and olfactory bulbs (OB) which were approximately twice less than in the DG. These results are in agreement with the immunohistochemistry observations described above. Linear regression analysis of the data (Fig 3B) revealed the correlation between band intensities detected by antibodies that recognized total ArfGAP1 and those recognizing the brain specific isoform only (R^2^ = 0.84359). As ArfGAP1^B^ is the major ArfGAP1 isoform in total brain [2], our findings are in agreement with the idea that the B isoform is predominant in all brain regions examined.

**Fig 4:**
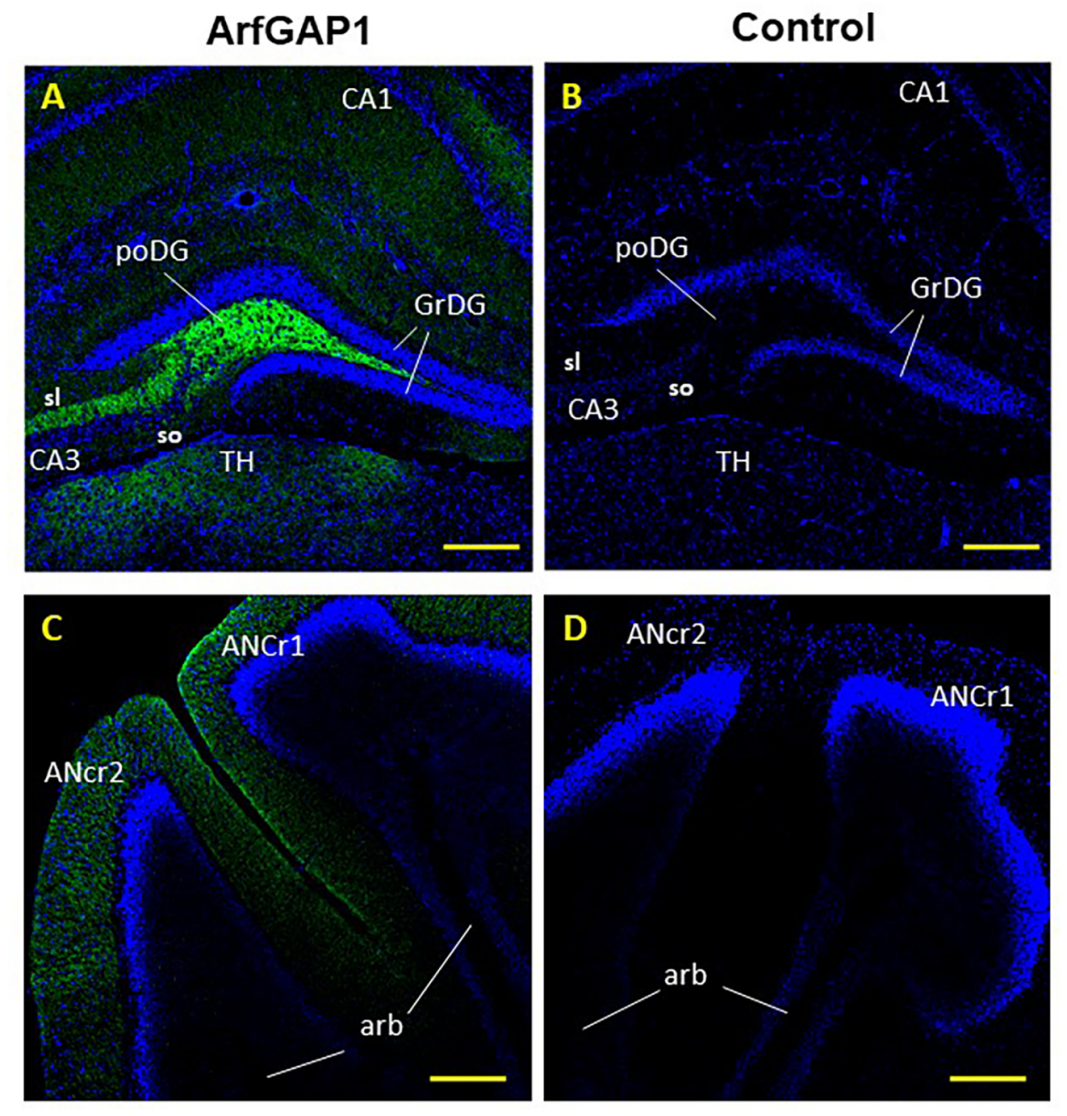
ArfGAP1 protein localization in mouse dentate gyrus and cerebelum. A, B. coronal view of the DG, poDG–polymorph layer (hilus) of the DG, grDG–granular cell layer of the DG, sr–stratum radiatum, slu–stratum lucidum, so–sorstratum oriens. CA1, CA2 and CA3 –Cornu Ammonis of the hippocampus. C, D. Coronal view of the cerebellum, ANcr1 and ANcr2 –ansiform cruciform lobule 1 and 2, arb–arbor vitae; ArfGAP1 (green), DAPI stain (blue). B,D–control experiments with the omission of primary antibody and staining only with secondary antibody. Magnification 10X. The scale bars represent 210 μm.

Despite identified difference between ArfGAP1 levels in the Hippocampus (in the DG area) and Cer, both brain regions have anatomically similar axons—mossy fibers (Fig 4). However, the functional properties of mossy fibers are different in each region [6]. Thus, our findings of differential expression of ArfGAP1 is probably related to functional properties of the axons. It is recognized that the hilus contains mossy cells and mossy fibers which represent the main part of this region, as well as interneurons and astrocytes [7]. The pattern of ArfGAP1 staining in this region (Fig 2) is similar to the mossy fibers projections usually seen via Timm's sulfide silver staining method [8, 9] which suggests that ArfGAP1 follows the path of mossy fibers—unmyelinated axons of the granule cells. It is known that mossy fibers give rise to a distinctive set of collaterals that heavily innervate cells within the hilus and have large terminals that form en passant synapses with the mossy cells in the hilus and with the CA3 pyramidal cells [10].

**Fig 5:**
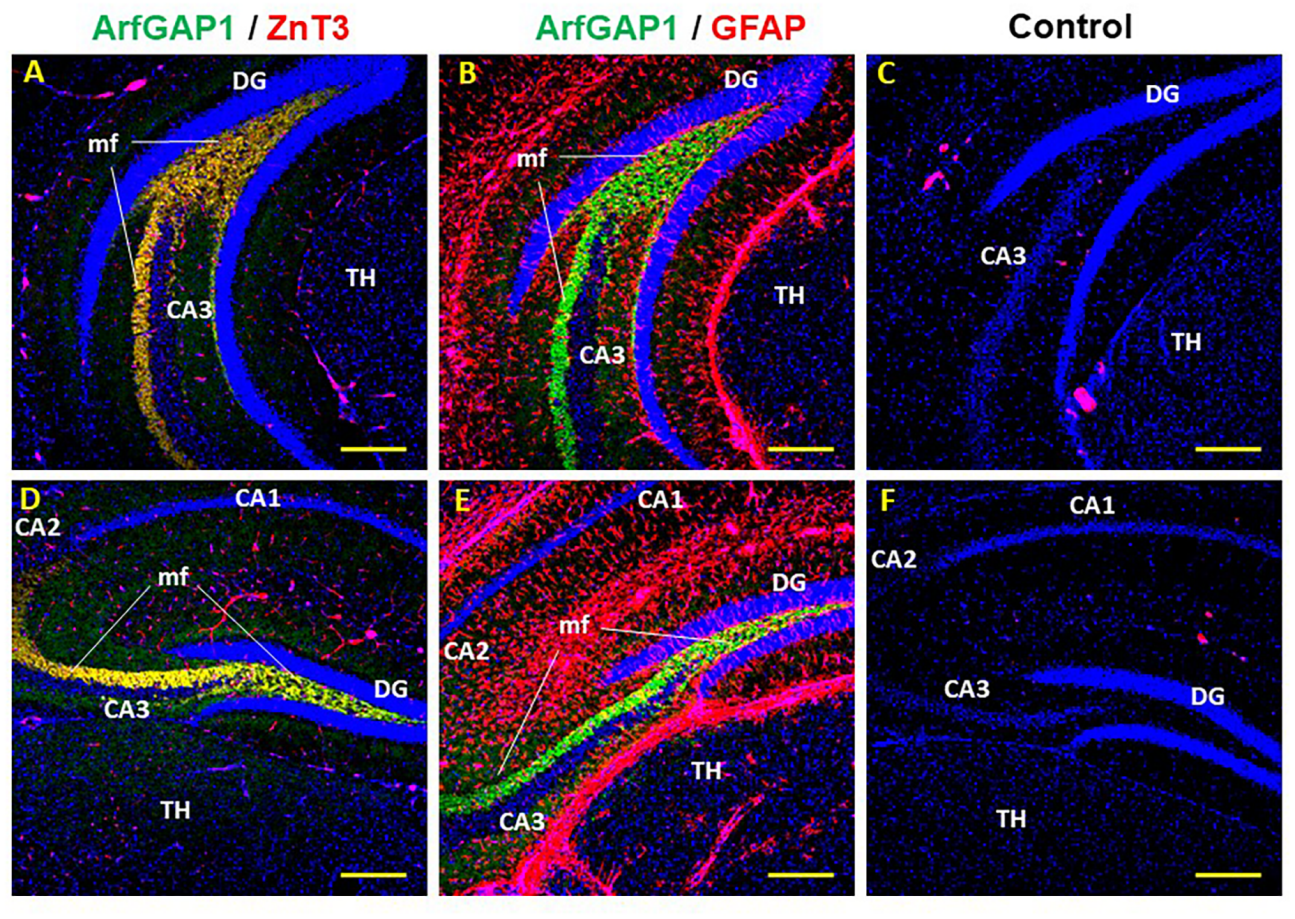
ArfGAP1 is localized in the mossy fibers of the mouse DG. A, B. Double labeling of ArfGAP1(green) and ZnT3 (red). Yellow staining indicates colocalization of ArfGAP1 and ZnT3. C, D. Double labeling of ArfGAP1(green) and GFAP (red). F, E. Omission of the primary antibody and staining only with secondary antibody. Abbreviations: CA1, CA2 and CA3 –Cornu Ammonis of the hippocampus. Coronal DG (dentate gyrus) view of ventral (A, C, F) and dorsal (B, D, E) hippocampus. TH–thalamus. mf–mossy fibers. Magnification 10X.The scale bars represent 210 μm.

To further demonstrate the concentration of ArfGAP1 in mossy fibers of the hilus, we carried out colocalization analysis using anti-zinc transporter 3 (ZnT3) antibodies—a known marker for mossy fibers [11] and anti-glial fibrillary acidic protein (GFAP) as a marker for astrocytes [12]. We observed colocalization of the ArfGAP1 fluorescence with that of ZnT3 in dorsal and ventral regions of the hippocampus (Fig 5A and 5B), whereas there was no colocalization between the astrocyte marker GFAP and ArfGAP1 in the hilus (Fig 5C and 5D). Moreover, similar to ZnT3, ArfGAP1 did not highlight the mossy cells and interneuron cell bodies. The distribution of ArfGAP1-immunoreactive mossy fibers projected uniformly along the dorsoventral axis of the hilus, in agreement with the other data described above (Figs 1 and 2). It is recognized that the pattern of ZnT-3 immunoreactivity resembles that observed with the Timm's stain since they both detect zinc-enriched mossy fibers [8,9,11]. However, the complexity of Timm's staining technique become a limiting factor to its applications for the studies using immunofluorescence. Our finding that ArfGAP1 is enriched in the Dentate mossy fibers adds a new perspective to use this antibody in immunofluorescence.

According to the Genome-wide atlas of gene expression in the adult mouse brain, the highest abundance of the ArfGAP1 mRNA are found in the granule cells of the DG, pyramidal cells of the hippocampus including *Cornu Ammonis* (CA) areas: CA1, CA2 and CA3 and partially in the cortex layer 1 [13].

Since ArfGAP1 translocates in cultured cells between cytosol and the Golgi complex, we expected to detect the protein in the soma or dendrites. However, we did not find any specific intracellular staining. We speculate that after expression in the DG granular cells, ArfGAP1 might translocate from the soma to the axon and accumulate there.

Overall, our findings indicate that ArfGAP1 can be used as a specific marker of the Dentate mossy fibers in immunohistochemical applications. Moreover, the high level of ArfGAP1 in the hilus of the DG point to a possible regulatory role of this protein in neurotransmission. This hypothesis is further suggested by the fact that ArfGAP1 was identified as an interaction partner of GABA transporter-1 that controls the recruitment of the transporter into transport vesicles and its trafficking through the secretory pathway to the membrane in axon terminals [3]. Interestingly it was speculated that in DG the mossy fiber terminals could co-release of both GABA and glutamate [14]. However, the possible role of ArfGAP1 in the regulation of trafficking of both neurotransmitters is unknown and required further investigations. Finally, our findings suggest that focusing on processes in the mossy fibers, in particular, may yield better understanding of the function of ArfGAP1 in brain.

## References

[1] Cukierman E, Huber I, Rotman M, Cassel D. The ARF1 GTPase-activating protein: zinc finger motif and Golgi complex localization. 1995, Science (New York, NY).270(5244):1999–2002. 10.1126/science.270.5244.1999 8533093

[2] Parnis A, Rawet M, Regev L, Barkan B, Rotman M, Gaitner M, et al Golgi Localization Determinants in ArfGAP1 and in New Tissue-specific ArfGAP1 Isoforms. 2006, J Biol Chem.281(7):3785–92. doi: 10.1074/jbc.M508959200 16316994 10.1074/jbc.M508959200

[3] Reiterer V, Maier S, Sitte HH, Kriz A, Rüegg MA, Hauri H-P, et al Sec24- and ARFGAP1-Dependent Trafficking of GABA Transporter-1 Is a Prerequisite for Correct Axonal Targeting. 2008, J Neurosci.28(47):12453–64. doi: 10.1523/JNEUROSCI.3451-08.2008 19020038 10.1523/JNEUROSCI.3451-08.2008 PMC4503341

[4] Candiello E, Kratzke M, Wenzel D, Cassel D, Schu P. AP-1/sigma1A and AP-1/sigma1B adaptor-proteins differentially regulate neuronal early endosome maturation via the Rab5/Vps34-pathway. 2016, Scientific reports.6:29950 doi: 10.1038/srep29950 27411398 10.1038/srep29950 PMC4944158

[5] Swenson R. Review of Clinical and Functional Neuroscience: Dartmouth Medical School; 2006. Available from: https://www.dartmouth.edu/~rswenson/NeuroSci/.

[6] Delvendahl I, Weyhersmuller A, Ritzau-Jost A, Hallermann S. Hippocampal and cerebellar mossy fibre boutons—same name, different function. 2013, J Physiol.591(13):3179–88. doi: 10.1113/jphysiol.2012.248294 23297303 10.1113/jphysiol.2012.248294 PMC3717221

[7] Amaral DG, Scharfman HE, Lavenex P. The dentate gyrus: fundamental neuroanatomical organization (dentate gyrus for dummies). 2007, Prog Brain Res.163:3–22. doi: 10.1016/S0079-6123(07)63001-5 17765709 10.1016/S0079-6123(07)63001-5 PMC2492885

[8] Wiera G, Mozrzymas JW. Extracellular proteolysis in structural and functional plasticity of mossy fiber synapses in hippocampus. 2015, Frontiers in Cellular Neuroscience.9(427). 10.3389/fncel.2015.00427 PMC4631828 26582976

[9] Sluyter F, Jamot L, Bertholet J-Y, Crusio WE. Prenatal exposure to alcohol does not affect radial maze learning and hippocampal mossy fiber sizes in three inbred strains of mouse. 2005, Behavioral and Brain Functions.1(1):5 doi: 10.1186/1744-9081-1-5 15916699 10.1186/1744-9081-1-5 PMC1143778

[10] Buchwalow I, Samoilova V, Boecker W, Tiemann M. Non-specific binding of antibodies in immunohistochemistry: fallacies and facts. 2011, Scientific reports.1:28 doi: 10.1038/srep00028 22355547 10.1038/srep00028 PMC3216515

[11] Palmiter RD, Cole TB, Quaife CJ, Findley SD. ZnT-3, a putative transporter of zinc into synaptic vesicles. 1996, Proc Natl Acad Sci U S A.93(25):14934–9. 8962159 10.1073/pnas.93.25.14934 PMC26240

[12] Orr AG, Hsiao EC, Wang MM, Ho K, Kim DH, Wang X, et al Astrocytic adenosine receptor A2A and Gs-coupled signaling regulate memory. 2015, Nat Neurosci.18(3):423–34. doi: 10.1038/nn.3930 25622143 10.1038/nn.3930 PMC4340760

[13] Lein ES, Hawrylycz MJ, Ao N, Ayres M, Bensinger A, al e. Genome-wide atlas of gene expression in the adult mouse brain. 2007, Nature.445(7124):168–76. doi: 10.1038/nature05453 17151600 10.1038/nature05453

[14] Münster-Wandowski A, Gómez-Lira G, Gutiérrez R. Mixed neurotransmission in the hippocampal mossy fibers. 2013, Front Cell Neurosci.7:210 doi: 10.3389/fncel.2013.00210 24319410 10.3389/fncel.2013.00210 PMC3837298

